# A Near InfraRed Emissive Chemosensor for Zn^2+^ and Phosphate Derivatives Based on a Di-(2-picolyl)amine-styrylflavylium Push-Pull Fluorophore

**DOI:** 10.3390/s23010471

**Published:** 2023-01-01

**Authors:** Liliana J. Gomes, João P. Carrilho, Pedro M. Pereira, Artur J. Moro

**Affiliations:** 1 LAQV-REQUIMTE, Departamento de Química, CQFB, Universidade Nova de Lisboa, 2829-516 Caparica, Portugal; 2 Intracelular Microbial Infection Biology, Instituto de Tecnologia Química e Biológica António Xavier, Universidade Nova de Lisboa, 2780-157 Oeiras, Portugal

**Keywords:** Near InfraRed fluorescent sensor, styrylflavylium, di-(2-picolyl)amine, zinc binding, adenosine 5′-triphosphate detection

## Abstract

A new Near InfraRed (NIR) fluorescent chemosensor for metal ions and anions is herein presented. The fluorophore is based on a styrylflavylium dye, a synthetic analogue of the natural anthocyanin family, with a di-(2-picolyl)amine (DPA) moiety as the metal chelating unit. The substitution pattern of the styrylflavylium core (with tertiary amines on positions 7 and 4′) shifts the optical properties of the dye towards the NIR region of the electronic spectra, due to a strong push-pull character over the π-conjugated system. The NIR chemosensor is highly sensitive to the presence of Zn^2+^, which induces a strong CHelation Enhanced Fluorescence (CHEF) effect upon binding to the DPA unit (2.7 fold increase). The strongest competing ion is Cu^2+^, with a complete fluorescence quenching, while other metals induce lower responses on the optical properties of the chemosensor. Subsequent anion screening of the Zn^2+^-chemosensor coordination compound has demonstrated a distinct selectivity towards adenosine 5′-triphosphate (ATP) and adenosine 5′-diphosphate (ADP), with high association constants (K ~ 10^6^ M^−1^) and a strong CHEF effect (2.4 and 2.9 fold fluorescence increase for ATP and ADP, respectively). Intracellular studies with the Zn^2+^-complexed sensor showed strong luminescence in the cellular membrane of Gram^–^ bacteria (*E. coli*) and mitochondrial membrane of mammalian cells (A659), which highlights its possible application for intracellular labelling.

## 1. Introduction

Fluorescent chemosensors have been widely investigated over the past decades, mainly due to their high sensitivity when compared to other sensor systems, allowing for a high spatio-temporal resolution and continuous monitoring of analyzed samples [1,2]. Over more recent years, a significant research effort has been directed to designing chemosensors with optical properties in the Near InfraRed (NIR) region, i.e., with absorption and emission above 650 nm. This specific class of sensors is particularly relevant for biological samples, since it is in this spectral region that light can penetrate deeper into cellular tissues, thus allowing in-vivo monitoring with minimal radiation damage [3]. Many systems have already been developed to target biologically important analytes, of which metal cations and anions represent the vast majority of reported works [4], although a strong emphasis has been directed also towards other relevant biomolecules such as enzymes or proteins [5].

Strategies for targeting metal cations generally involve coupling fluorophores with metal chelating moieties, which can be bound directly or via a spacer [6]. Amongst the many known binding units for metal cations, di-(2-picolyl)amine (DPA) emerges as one of the most commonly employed, as it has been widely reported to preferentially bind to Zn^2+^ cations, usually yielding a CHelation Enhancement of Fluorescence (CHEF) which is highly desirable to discard artefacts such auto-fluorescence or inner-filter effects (typically associated with emission quenching) [7,8,9]. Nevertheless, several systems based on DPA have also been developed for selective detection of Cu^2+^, and most of these sensors exhibit CHElation induced Quenching (CHEQ) [10].

The major breakthrough for DPA arose from the reports by Hamachi and co-workers, who used fluorescent Zn^2+^-DPA coordination compounds to detect phosphorylated peptides and other phosphate derivatives [11,12]. These pioneering works opened a whole sub-field of fluorescent sensors, directed to the detection of anionic species [13,14,15,16].

**Figure 1:**
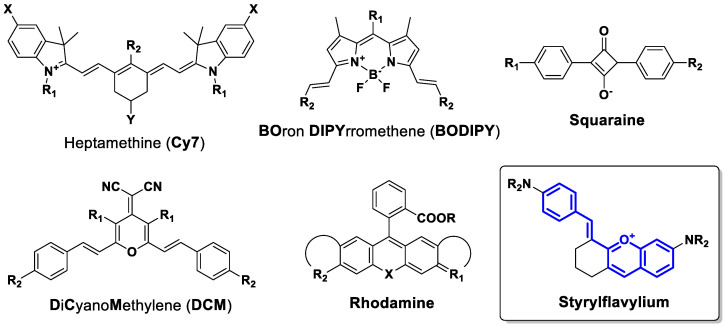
Examples of NIR fluorophores. The styrylflavylium core is highlighted in blue.

With respect to NIR fluorophores, some of the most noteworthy include heptamethyne cyanine [17], BODIPYs [18], squaraine [19,20], dicyanomethylene [21,22] and modified rhodamines [23,24] (Figure 1). Although not regarded as your typical fluorescent unit, synthetic flavylium dyes can be easily modified to include moieties for binding external analytes, thereby allowing for the development of new probes [25,26,27]. Furthermore, extension of the π-conjugated benzopyrilium core is readily achieved with the appropriate precursors to produce styrylflavylium derivatives [28,29] (Figure 1, blue). Indeed, these styrylflavylium dyes have been recently reported as red or NIR fluorescent probes for hydrogen sulfide [30], sulfur dioxide [31], hydrazine [32] and sensing DNA replication [33]. With this in mind, we designed a novel styrylflavylium chemosensor bearing a DPA receptor, and assessed its potential as a sensor for cations and anions.

## 2. Experimental Section

### 2.1. Synthesis

All used chemicals were of analytical grade and used as purchased. Fine chemicals were acquired from Sigma-Aldrich (St. Louis, MO, USA), while solvents were purchased either from Sigma-Aldrich or Carlo Erba (Barcelona, Spain).

Synthesis of 4-(4-((2-(bis(pyridin-2-ylmethyl)amino)ethyl)(methyl)amino)benzylidene)-6-(diethylamino)-1,2,3,4-tetrahydroxanthylium (**1**)

In a 25 mL round-bottom flask, **A** (1 eq, 200 mg, 0.45 mmol) and 4-(Diethylamino) salicylaldehyde (1 eq, 87.7 mg, 0.45 mmol) were dissolved in 6 mL of acetic acid. Later, under vigorous stirring, a solution of sulfuric acid (1.5 mL) was added dropwise, immediately turning into a dark purple color. The reaction was followed by TLC using as eluent hexane:ethyl acetate (7:3) until the complete consumption of the starting material (~24h). The final reaction mixture had a dark blue/purple color. The solvent was removed under reduced pressure, and the reaction mixture was dried under vacuum. Afterwards, the crude was purified by RP18 column chromatography starting with HCl 0,1 M:MeOH 75:25 as eluent and gradually changing its polarity until 100% methanol. The collected fractions were analyzed by HPLC to identify the product fractions (following absorption at 670 nm), and the solvents were removed under reduced pressure. Finally, 170 mg of **1** were obtained as a dark blue oil (η= 62.54%), which was further washed with ether and centrifugated to obtain a dark blue precipitate. ^1^H RMN (400 MHz, MeOD) δ (ppm): 8.31 (d, *J* = 5.6 Hz, 2H), 8.06 (t, *J* = 7.6 Hz, 2H), 7.88 (s, 1H), 7.65 (d, *J* = 7.7 Hz, 2H), 7.57 (s, 1H), 7.50 (t, *J* = 6.7 Hz, 2H), 7.36 (d, *J* = 9.4 Hz, 1H), 7.23 (q, *J* = 8.4 Hz, 4H), 6.93 (d, *J* = 9.4 Hz, 1H), 6.79 (s, 1H), 3.82 (s, 4H), 3.61 (t, *J* = 6.4 Hz, 2H), 3.24 (q, *J* = 7.0 Hz, 4H), 2.77 (s, 3H), 2.43 (q, *J* = 5.5 Hz, 6H), 1.43 (q, *J* = 5.8 Hz, 2H), 0.82 (t, *J* = 6.9 Hz, 6H). ^13^C NMR (101 MHz, MeOD) δ (ppm): 163.04, 160.73, 158.23, 153.38, 149.84, 148.64, 143.01, 142.53, 137.83, 134.97, 133.94, 133.51, 131.35, 129.12, 127.86, 125.33, 122.14, 121.64, 97.00, 66.91, 56.69, 55.58, 50.84, 47.48, 46.82, 28.36, 28.17, 22.71, 12.97 (all collected spectra and peaks assignments are found in the Supporting Information, Figures S1–S5 and Table S1). HR-ESI-MS (+) m/z: 598.3547 ([M + H]+, calcd: 598.3541) (Figure S6).

### 2.2. UV-Vis and Fluorescence Measurements

Solutions for UV-Vis absorption and fluorescence measurements were prepared by adding an aliquot of 30 µL of a 3 × 10^−4^ M methanolic solution of flavylium **1**, to 1470 µL of methanol, and 1100 µL of aqueous buffer, for a final chemosensor concentration of 1 µM. For all metal titrations, 10 mM 3-(*N*-morpholino)propanesulfonic sodium salt (MOPS) buffer at pH 7.0 ± 0.2 was used. pH titrations were performed using Theorell and Stenhagen universal buffer [34]. Metal ion titrations were performed by adding small aliquots of solution containing **1** and each of the studied metals to a cuvette containing solely chemosensor **1**, to ensure that the concentration of the latter remained constant. Both UV-Vis as well as luminescence spectra were recorded in between additions. The limit of detection (LOD) and limit of quantification (LOQ) of Zn^2+^ were determined according to IUPAC guidelines [35], by measuring five independently prepared samples of styrylflavylium **1** with no metal (blank) and applying the formulae: LOD = 3*σ*/*b* and LOQ = 10 *σ*/*b*, where *σ* represents the standard deviation of these measurements, and *b* represents the slope over a fixed linear range (0–3µM was selected). Anion titrations were performed in a similar manner but using a cuvette containing **1**-Zn^2+^ as the starting point, to which aliquots of solution containing **1**-Zn^2+^ and anion were successively added. Absorption spectra were acquired in a 1 cm quartz cuvette on a Varian Cary 100 Bio UV- spectrophotometer. Emission spectra were obtained in a 1 cm fluorescence quartz cuvette, using a Horiba-Jobin-Yvon SPEX Fluorolog 3.22 spectrofluorometer. Fluorescence quantum yield for **1** was determined using Cryptocyanine as reference (ϕ_f_ = 0.007 in ethanol) [36]. The binding constants for **1**-Zn^2+^, (**1**-Zn^2+^)-ATP and (**1**-Zn^2+^)-ADP were determined by fitting the experimental data to a Henderson-Hasselbalch 1:1 binding model using the Solver Add-In from Microsoft Excel [37].

#### 2.2.1. Minimum Inhibitory Concentration (MIC) Determination

The MICs of **1** for *Staphylococcus aureus* (*S.* aureus) strain JE2 (Community-acquired Methicillin Resistant *S. aureus*, CA-MRSA) and *Escherichia coli* (*E. coli)* strain DC10B were determined in sterile 96-well microplates using Muller Hinton Broth (MHB) or Luria Broth (LB), respectively, supplemented with 4 equivalents of Zn^2+^ (ZnSO_4_) for each concentration of **1**. **1** was added to each microplate well to obtain 2-fold serial dilutions of the compound with the highest concentration being 32 μg/mL. Cultures of *S. aureus* and *E. coli* were added to the wells at a final density of 5 × 10^5^ CFU/mL. A number of wells were reserved in each plate for sterility control (no inoculum added) and inoculum viability (no **1** added). Plates were incubated at 37 °C and growth was recorded visually at 24 and 48 h. All MICs were determined in triplicate for each condition assayed.

#### 2.2.2. Biocompatibility Assay in A549 Lung Carcinoma Cells

A549 lung carcinoma cells were seeded (1 × 10^5^) in 8-well slides and incubated in DMEM containing 10% FBS, 100 U/mL penicillin and 100 μg/mL streptomycin at 37 °C with 5% CO_2_ in a humidified incubator for 24 h. Cells were subsequently incubated for 24 h in the presence or absence of **1** (2 and 4 μg/mL) supplemented with 4 equivalents of Zn^2+^ (ZnSO_4_). Upon 24 h incubation cells were washed (with 1X Phosphate-buffer Saline, PBS) to removed dead cells, incubated in Trypsin/EDTA solution (0.05% trypsin, 0.53 mM EDTA) and enumerated using a cell drop (DeNovix) in triplicate, following manufactures recommendations.

#### 2.2.3. Microscopy Experiments

For microscopy experiments with A549 cells (European Collection of Authenticated Cell Cultures (ECACC), #86012804 [38]. Cells were grown in DMEM containing 10% FBS, 100 U/mL penicillin and 100 μg/mL streptomycin at 37 °C with 5% CO_2_ in a humidified incubator. Before imaging cells were seeded at 1 × 10^5^ on to 8-well slides and grown for 24 h. **1** was added at 4 μg/mL (with 4 equivalents of Zn^2+^, ZnSO_4_) and cells were imaged for up to 16 h at 37 °C with 5% CO_2_ in a humidified microscope incubator (Pecon).

For microscopy experiments with *S. aureus* JE2 [39] strain and *E. coli* DC10B strain [40] bacterial cells. Bacteria were grown in MHB or LB, respectively, until mid-exponential phase (OD_600nm_ of 0.5). 1 mL of cells was pelleted (13.000 RPM, 1 min) and subsequently resuspended in 10 mM MOPS with **1** at 4 μg/mL (with 4 equivalents of Zn^2+^, ZnSO_4_) and incubated for 5 min at 23 °C. Cells were pelleted (13.000 RPM, 1 min), resuspended in 10 μL, and 2 μL were mounted on a thin layer of 1X PBS with 1.2% agarose, covered with a 1.5H coverslip, and imaged.

Imaging was performed on a motorised inverted widefield (WF) fluorescence microscopy system (Axio Observer 7, Zeiss) equipped with Colibri 7 LED illumination (Zeiss), with excitation LED of 653 nm and emission filter EM BP 681/45 (Filter set 90, Zeiss), using a Plan-Apochromat 63×/1.4 oil objective (Zeiss). Focus during experiments was kept using the definite focus 3 (Zeiss). Images were captured using a Prime PB 95B (Photometrics) camera using ZEN software (Blue edition, SW ZEN 3.4 pro).

## 3. Results and Discussion

### 3.1. Synthesis and Photophysical Characterization

**Figure 2:**
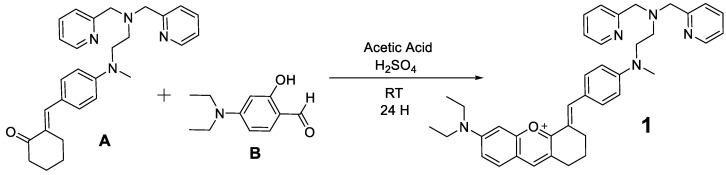
Synthesis reaction for styrylflavylium **1**.

Chemosensor **1** was designed to possess the flavylium chromophoric unit coupled to a DPA metal chelating moiety. The synthesis was performed by condensation of (E)-2-(4-((2-(bis(pyridin-2-ylmethyl)amino)ethyl)(methyl)amino)benzylidene)cyclohexanone (**A**) [41] with 4-(diethylamino)salicylaldehyde (**B**) allowed to obtain styrylflavylium **1** (Figure 2).

**Figure 3:**
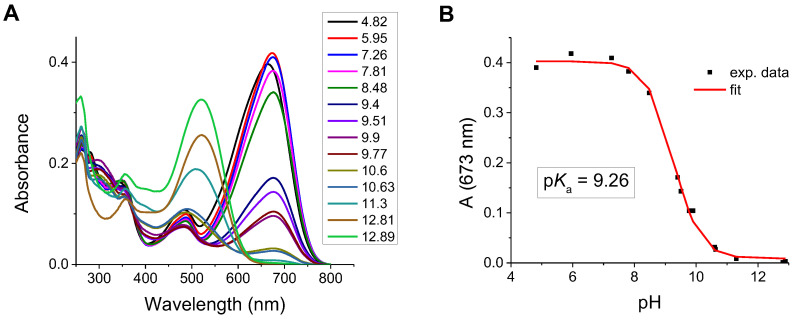
(**A**) UV-Vis absorption spectra of **1** in neutral to basic conditions (4.8 < pH < 12.9); (**B**) experimental data points collected at 673 nm, with corresponding fit for the pH equilibrium. Experimental conditions: [1] = 1.7 × 10^−5^ M, in MeOH:Theorell and Stenhagen universal buffer:H_2_O 50:25:25.

Flavylium and styrylflavylium salts are involved in a multi-state equilibrium which is determined by the pH of the medium. The molar fraction of each chemical species at the different pH values/ranges is highly dependent on the substitution pattern of the flavylium core [27]. Furthermore, the energetic barrier associated with the isomerization process, from the hemiketal species **B** to the *cis*-chalcone **Cc** may also play a key role [25]. As such, we performed pH titrations of **1**, in order to get a general overview of the species present at the different pH values (Figure 3 and Figure S7).

**Figure 4:**
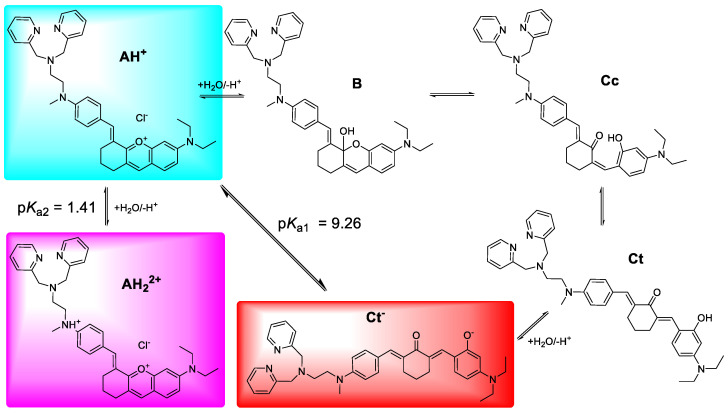
(A) UV-Vis absorption spectra of **1** in neutral to basic conditions (4.8 < pH < 12.9). Multi-state equilibria for 1, at different pH values. Styrylflavylium (AH+, blue) is stable in moderately acid to neutral pH.

The pH titration shows that, at moderately acid conditions (pH~4), the main species present is the styrylflavylium cation **AH^+^** (blue in Figure 4) with an absorption maximum at around 670 nm. As the pH increases, the equilibrium is shifted towards the formation of the trans-chalcone species in its deprotonated state, **Ct^–^** (red in Figure 4) revealed by the appearance of a new band at around 530 nm. The overall p*K*_a_ of this transformation (p*K*_a1_) was determined to be at pH = 9.26, which tells us that the styrylflavylium is suitable for sensing applications within the physiological pH range.

At extremely acidic conditions, it is possible to observe the protonation of the styrylflavylium to form **AH_2_^2+^** (violet in Figure 4), with a p*K*_a2_ value of 1.41 (Figure S7B). All other species are not observable at equilibrium conditions, indicating that they exist as transient species as observed in other similar systems [28,29,42].

The fluorescence quantum yield of **1** was determined to be 0.003 (cryptocyanine was used as reference [36]), with a large Stokes shift (~75 nm, 1460 cm^−1^). These values indicate the presence of a deactivation mechanism related to Planar Internal Charge Transfer (PICT), as suggested previously for the 7-(*N*,*N*-diethylamino)-4′-hydroxy-flavylium analogue [43].

### 3.2. Metal Sensitivity/Selectivity

**Figure 5:**
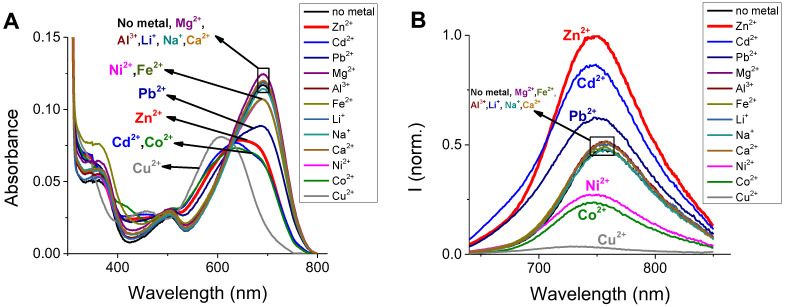
(**A**) UV-Vis absorption spectra and (**B**) emission spectra of **1** (5 µM), in the presence of different metal cations (10 µM). Conditions: MeOH:MOPS 10 mM pH7 (50:50); λ_exc_ = 620 nm.

Styrylflavylium **1** comprises a di-(2-picolylamine) moiety (DPA), capable of binding metal cations [6]. A screening of mono- and divalent cations was performed to assess the potential selectivity and the effect of the binding event in the optical properties of **1**, by adding two equivalents of each cation and monitoring the changes in UV-Vis absorption and fluorescence spectra (Figure 5).

In the UV-Vis spectra (Figure 5A), we can observe that several cations induce a hypochromic shift on the absorption of **1**, namely Ni^2+^, Fe^2+^, Pb^2+^, Zn^2+^, Cd^2+^, Co^2+^ an Cu^2+^, with this latter ion being the one that exhibits the strongest effect, with the original absorption band disappearing and a new band rising at *circa* 600 nm of absorption maximum. In the emission (Figure 5B), the panorama is quite different. Indeed, while Ni^2+^, Co^2+^ and Cu^2+^ promote fluorescence quenching, Zn^2+^, Cd^2+^ and Pb^2+^ (this latter to a smaller extent) induce an enhancement in the emission of **1**. This effect is similar to previously reported fluorescent sensor molecules bearing DPA units [44,45,46,47,48,49,50].

It is therefore noteworthy that the ions that induce the strongest changes are Zn^2+^ and Cu^2+^, even though their effect upon binding to **1** is opposite when looking at the emission. To better understand the differences in binding strength, independent titrations of **1** with each of these two metals were made.

**Figure 6:**
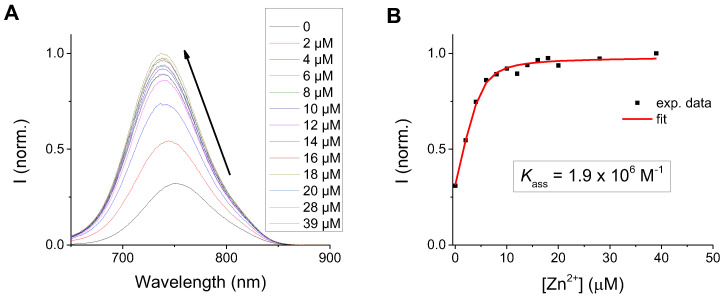
(**A**) Emission spectra and (**B**) corresponding changes in the total emission of **1** (5 µM), in the presence of increasing amounts of Zn^2+^. Conditions: MeOH:MOPS 10 mM pH7 (50:50); λ_exc_ = 620 nm.

Addition of gradual amounts of Zn(II) to a solution of **1** led to a decrease in the UV-Vis absorption spectra, with a hypsochromic shift of the absorption maximum, from ~680 to ~660 nm (Figure S8). These changes in the absorption of **1** suggest that the nitrogen in position 4′ of the styrylflavylium core is also participating in the coordination of the metal ion (Figure S9). In terms of emission, the luminescence of **1** exhibited CHelation Enhanced Fluorescence (CHEF), with a 2.7 fold increase and a slight shift to shorter wavelengths (~15 nm), similar to the corresponding absorption spectra (Figure 6A).

**Figure 7:**
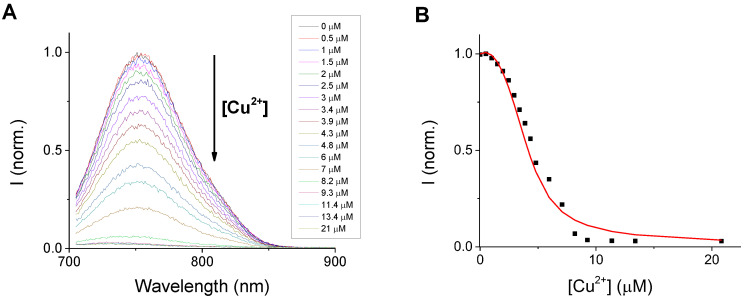
(**A**) Emission spectra and (**B**) corresponding changes in the total emission of **1** (5 µM), in the presence of increasing amounts of Cu^2+^. The red line represents the best fit with the experimental data (*K*_1_ = 4.97 × 10^6^ M^−1^ and *K*_2_ = 1.15 × 10^5^ M^−1^). Conditions: MeOH:MOPS 10 mM pH7 (50:50); λ_exc_ = 680 nm.

The binding constant was obtained by fitting the experimental data to a 1:1 binding model, yielding a value of 1.9 × 10^6^ M^−1^ (Figure 6B). This value is in line with previously reported DPA sensor systems for divalent ions [7,8,9,10]. As a comparison, the same experiments were also performed for Cd^2+^, since it exhibited a similar behavior as Zn^2+^ (see Figure 5B), resulting in a lesser fluorescence enhancement and a binding constant 4 times lower than that of Zn^2+^ (Figure S10). The limit of detection (LOD) and limit of quantification (LOQ) for Zn^2+^ were determined according to IUPAC guidelines (see Section 2) and were found to be 0.39 µM and 1.29 µM, respectively. On the other hand, Cu^2+^ induced a much stronger response on the optical properties of chemosensor **1**. Indeed, the UV-Vis absorption spectra changes dramatically upon increasing Cu^2+^ concentrations, with a new band appearing at λ_max_ = 589 nm (Figure S11A), while the emission exhibits a CHElation induced Quenching effect (Figure 7A).

**Scheme 1:**
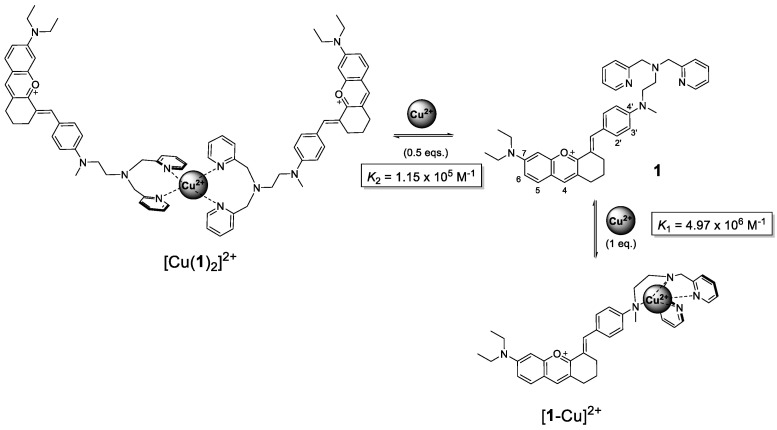
Proposed mechanisms for the coordination of Cu(II) with chemosensor **1**.

Interestingly, plotting the emission intensity (or the maximum absorbance) against the concentration of Cu^2+^ reveals the presence of a small plateau at the lowest metal concentration range, with the strongest changes appearing as we approach 1 equivalent of metal (Figure 7B and Figure S11B). This behavior suggests that, at an initial stage, the metal ion may be capable coordinating with the DPA units from two different fluorophore molecules without affecting the nitrogen in position 4′, which results in little effect on the optical properties of **1**. After 1 equivalent, an abrupt emission quenching and absorption change is observed, signaling a disruption in the π-conjugated system, which indicates the participation of the nitrogen in position 4′ in the coordination sphere with Cu(II) (Scheme 1). The data (absorption and emission) were fitted to an equilibrium comprising the formation of a 1:1 complex and a 2:1 complex (i.e., two units of chemosensor **1** binding to one Cu^2+^ ion), yielding association constants with values of *K*_1_ of 4.97 × 10^6^ M^−1^ and a *K*_2_ = 1.15 × 10^5^ M^−1^ (see Supporting Information for details). Despite this behavior, Job’s plot indicates a 1:1 binding stoichiometry with **1** for both Zn^2+^ and Cu^2+^ (Figure S12).

**Figure 8:**
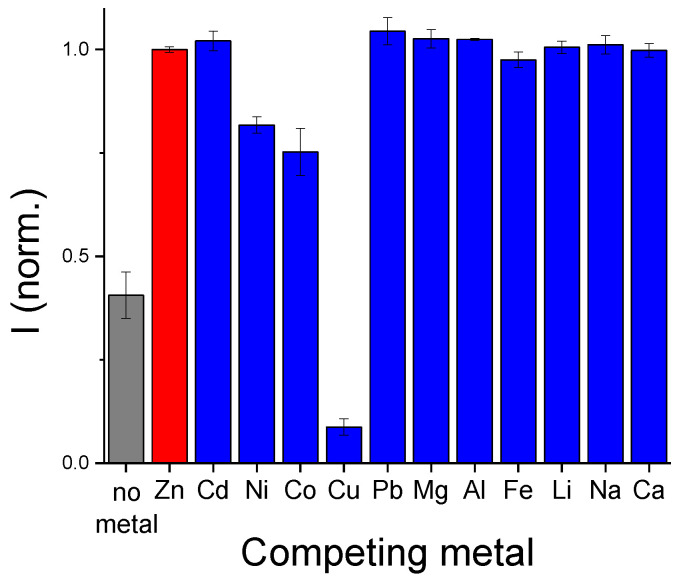
Fluorescence competition assay illustrating the influence of other metals (1 eq.) in the detection of Zn^2+^ ions (1 eq.) (blue). Emission intensity of **1** in the absence of any metal (grey) or in the presence of 1 eq. of Zn^2+^ (red) was added for comparison. Conditions: MeOH:MOPS 10 mM pH7 (50:50); λ_exc_ = 620 nm.

Since our focus was on applying this sensor system in biological applications and free intracellular Cu(II) concentration are extremely low, we decided to direct our efforts towards Zn(II). As such, we performed a competition assay in which 1 equivalent of each competing metal cation was added to solutions containing 1 equivalent of Zn^2+^, and the corresponding emission was recorded (Figure 8).

The results show that Cu(II) is, by far, the strongest interferent in Zn^2+^ detection with **1**, which is consistent with the higher association constant values obtained for this ion. Of the remaining studied metals, only Co^2+^ and Ni^2+^ induce some emission quenching (23% and 18% respectively) with respect to the emission of **1**-Zn^2+^, while the effect of other metal cations in the emission of **1** are much smaller or negligible (≤10%).

**Figure 9:**
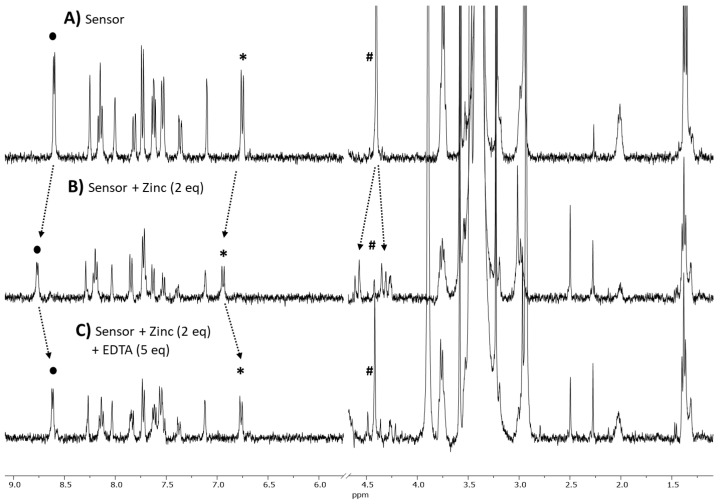
^1^H NMR spectra of [1] = 0.78 mM: (**A**) with no metal; (**B**) in the presence of 2 equiv. of zinc; (**C**) in the presence of 2 eqs. of zinc and 5 eqs. of EDTA. All spectra were conducted at room temperature in a mixture of MeOD:D_2_O (1:1).

In order to further evaluate Zn(II) coordination, NMR titration of **1** was carried out in MeOD:D_2_O (1:1) with zinc between 0 and 2 equivalents (Figure 9 and Figures S13 and S14). According to the results obtained in the NMR spectra, aromatic proton peaks at circa 8.6 and 6.7 ppm become gradually unshielded upon adding zinc, which translates to the appearance of new peaks at around 8.75 and 6.9 ppm (Figure 9). In addition, the singlet present at 4.4 ppm, corresponding to CH_2_ protons of the DPA unit, disappears in the presence of Zn^2+^, giving rise to two small doublets in the same spectral region. This observation is consistent with the rigidification of the DPA unit upon complexation with the metal, since these protons may behave like diastereotopic protons (Figure S9). Moreover, an additional experiment was performed, through the addition of excess (5 equivalents) of ethylenediamine tetraacetate (EDTA), a known complexing agent for Zn^2+^ and other divalent metals (Figure 9c) [51]. As we expected, the protons signals of free **1** in solution were regenerated, meaning that the complexation between sensor and metal is reversible in the presence of a stronger chelating agent.

#### 3.2.1. Anion Sensing

**Figure 10:**
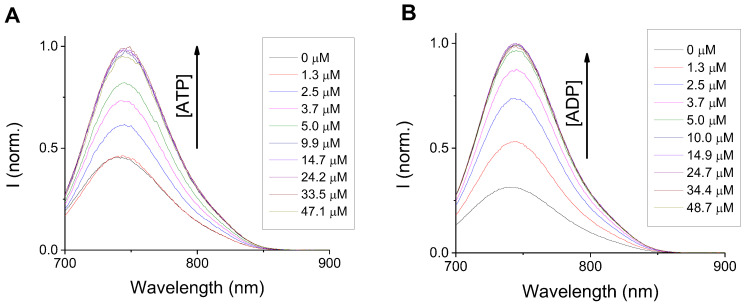
Emission spectra of **1**-Zn^2+^ with increasing concentration of (**A**) ATP and (**B**) ADP. Conditions: MeOH:MOPS 10 mM pH7 (50:50); λ_exc_ = 680 nm.

Many examples of metal-coordinated DPA fluorescent chemosensors are known to exhibit the ability to subsequently detect anionic species. In particular, Zn^2+^-DPA coordination compounds have been widely used for sensing of phosphate and phosphate derivatives [2,13]. As such, we screened the optical properties Zn(II)-coordinated chemosensor **1** (henceforth referred to as **1**-Zn^2+^) against a series of anions (Figure S15). We observed a strong selectivity of **1**-Zn^2+^ towards adenosine 5′-triphosphate (ATP) and adenosine 5′-diphosphate (ADP), with an emission enhancement of 2.1- and 3.0-fold, respectively. Out of the other studied anions, only citrate induced some emission enhancement (~0.36 fold), while pyrophosphate (PPi) induced a slight (~22%) emission quenching. The remaining anions exerted no effect on the emission of **1**-Zn^2+^. Fluorescence titrations with ATP and ADP fitted well to 1:1 binding stoichiometries and revealed a higher affinity towards ADP in comparison to ATP, with association constants of 6.3 × 10^7^ M^−1^ and 1.5 × 10^6^ M^−1^, respectively (Figure 10 and Figure S16).

The corresponding UV-Vis absorption spectra for both of these anions present an interesting feature. Indeed, as the concentration of ATP (or ADP) increases, we observe a slight bathochromic and hyperchromic shift, exactly opposite to the spectral behavior before Zn(II) complexation (Figure S17). This seems to indicate that binding the anion to the Zn(II) metal center causes the displacement of the nitrogen atom in position 4′ of the styryl flavylium core (Figure S18).

To confirm that the presence of Zn^2+^ is essential for anion binding, a simple test was performed. Briefly, fluorescence of **1** was recorded before and after the addition of 5 equivalents of ATP, with no changes in the acquired spectra (Figure S19). Upon subsequent addition of 2 equivalents of Zn^2+^, the emission from **1** registered the same behavior as previously shown in the presence of both metal and anion.

#### 3.2.2. Biocompatibility and Live-Cell Imaging Studies Using with Chemosensor **1**

To understand the viability of using chemosensor **1** for ATP localization, biocompatibility and WF live-cell microscopy studies were performed in mammalian cells (A549 lung carcinoma cells) and bacterial cells (*S. aureus* and *E. coli,* Gram positive (+) and negative (−) bacterial cells, respectively).

**Figure 11:**
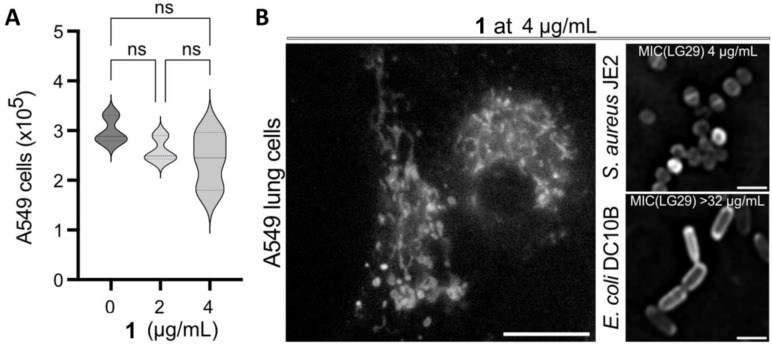
(**A**) A549 viability in the presence of increasing concentration of **1** for 24h. (**B**) WF live-cell imaging of A549 lung cells, *S. aureus* JE2 strain bacterial cells and *E. coli* DC10B strain bacterial cells stained with 4 μg/mL chemosensor **1** with 4 equivalents of Zn^2+^. Scale bars are 20 μm for A549 and 2 μm for *S. aureus* and *E. coli* images.

Incubation of A549 cells with concentrations of **1** up to 4 μg/mL revealed a high degree of biocompatibility, with a survival rate of this cell line, over a 24h time incubation period, comparable to untreated cells (Figure 11a). This was further confirmed by live-cell imaging microscopy experiments, where normal cell morphology and cell division events were observed during a 16h time-lapse in the presence of chemosensor **1** (movieS1). Fluorescence microscopy imaging showed that uptake of **1** resulted in a mitochondrial subcellular localization (Figure 11b), with no effect on mitochondrial dynamics (movieS2). The observed subcellular localization is consistent with the fact that ATP is significantly more concentrated in mitochondria [52].

To assess the viability of using chemosensor in bacterial cells we determined the MIC of **1** for two classical bacterial model organisms, the Gram (+) *S. aureus* and the Gram (−) *E. coli*. For *E. coli* DC10B strain (non-pathogenic lab strain) we observed no toxicity (MIC > 32 μg/mL, maximum concentration used). For *S. aureus* bacteria we observed that **1** has antimicrobial activity, with a MIC of 2 μg/mL. This observation is especially interesting considering the clinical importance of the JE *S. aureus* strain used in this assay, the most prominent CA-MRSA lineage in the United States [39]. For both bacterial models a homogeneous membrane associated localization was observed (Figure 11b), consistent with ATP synthesis in the bacterial membrane [52].

## 4. Conclusions

A new NIR emissive chemosensor system for the detection of cations was designed and fully characterized. Sensor **1** was capable of binding to several divalent metal ions through a di-(2-picolyl)amine (DPA) chelation moiety, presenting distinct spectral behaviors. UV-Vis spectral profile revealed participation of the nitrogen atom directly coupled to the fluorophore in the metal-coordination sphere. Of the studied metals, Zn^2+^ and Cd^2+^ induced a strong CHEF effect on **1**, with Zn^2+^ presenting a higher binding constant and Cu^2+^ acting as the strongest interferent. These results are comparable to previous works that use DPA as metal binding unit. Subsequent binding of ATP and ADP to **1**-Zn^2+^ was observed through a further fluorescence enhancement, with substitution of the ancillary nitrogen (position 4′) from the coordination sphere, a response which has been previously described in other works [47]. Intracellular studies indicate that **1**-Zn^2+^ may be employed to label bacteria and mammalian cells, particularly targeting the cellular membrane for the case of bacteria, and mitochondrial membranes from eukaryotic cells, due to the high concentrations of ATP/ADP in these intracellular sites.

## Data Availability

Not applicable.

## Supplementary Materials

The following supporting information can be downloaded at: https://www.mdpi.com/article/10.3390/s23010471/s1.
