# Supplementary material for: A Near InfraRed Emissive Chemosensor for Zn2+ and Phosphate Derivatives Based on a Di-(2-picolyl)amine-styrylflavylium Push-Pull Fluorophore
Source: Sensors (Basel). 2023 Jan 1;23(1):471. doi: 10.3390/s23010471 (PMC9823994; doi:10.3390/s23010471)
Supplement: Supplementary file 1 [file sensors-23-00471-s001.zip › Supporting Information.pdf]

SUPPORTING INFORMATION

A Near InfraRed emissive chemosensor for Zn^{2+} and phosphate derivatives based on a di-(2-picolyl)amine-styrylflavylium push-pull fluorophore

Liliana J. Gomes[a], João P. Carrilho[b], Pedro M. Pereira[b], Artur J. Moro* [a]

[a]*LAQV-REQUIMTE, Departamento de Química, CQFB, Universidade Nova de Lisboa, Monte de Caparica, Portugal.*

[b]*Intracelular Microbial Infection Biology, Instituto de Tecnologia Química e Biológica António Xavier, Universidade Nova de Lisboa, Oeiras, Portugal.*

* *E-mail: artur.moro@fct.unl.pt*

NMR spectra

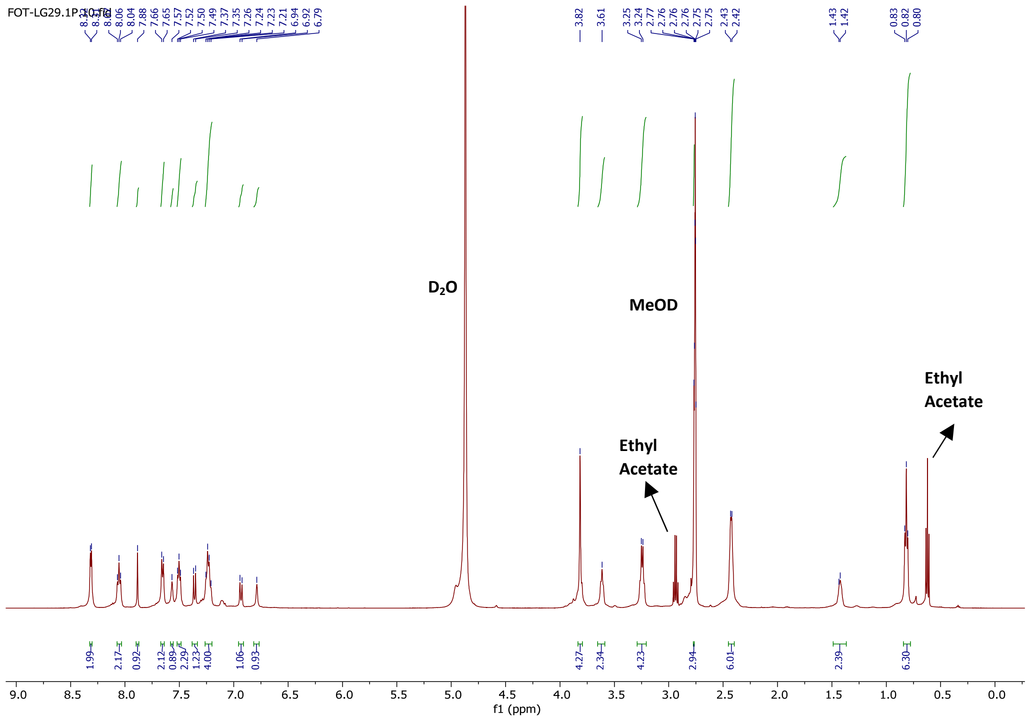

Figure S1. ^{1}H NMR spectrum of **1**.

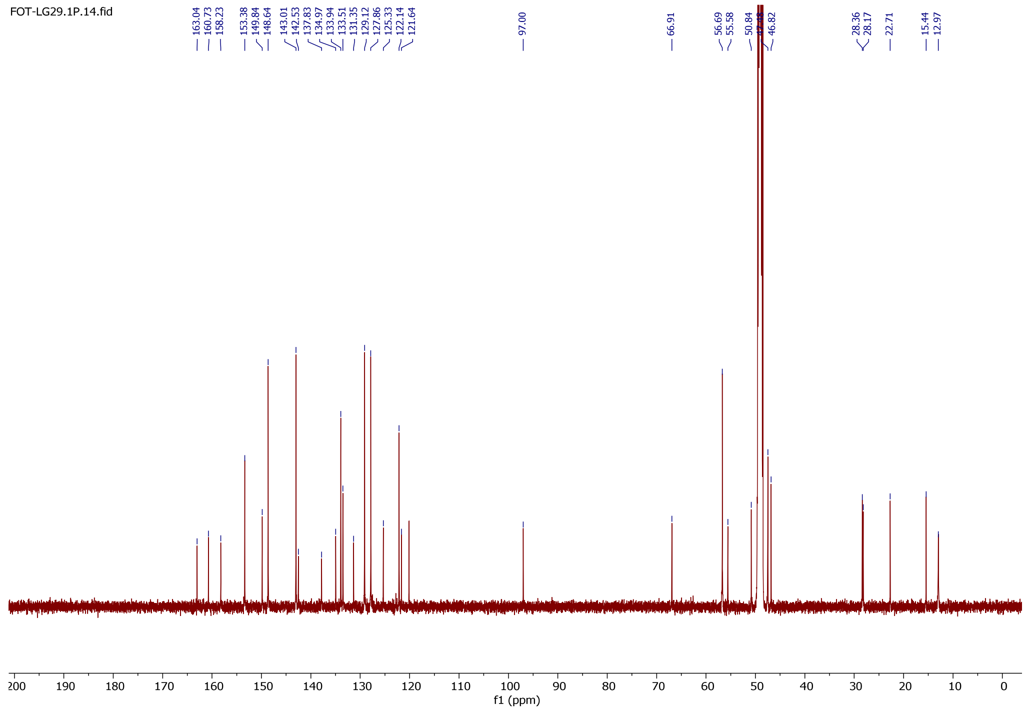

Figure S2. ^{13}C NMR spectrum of **1**.

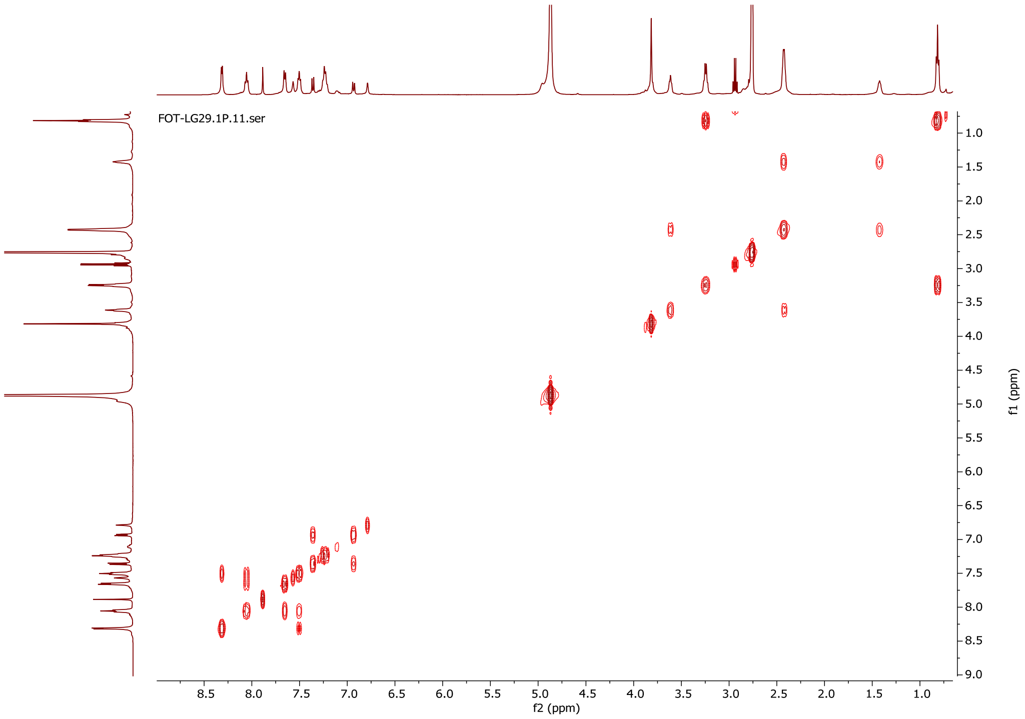

Figure S3. ^{1}H-^{1}H COSY NMR spectrum of **1**.

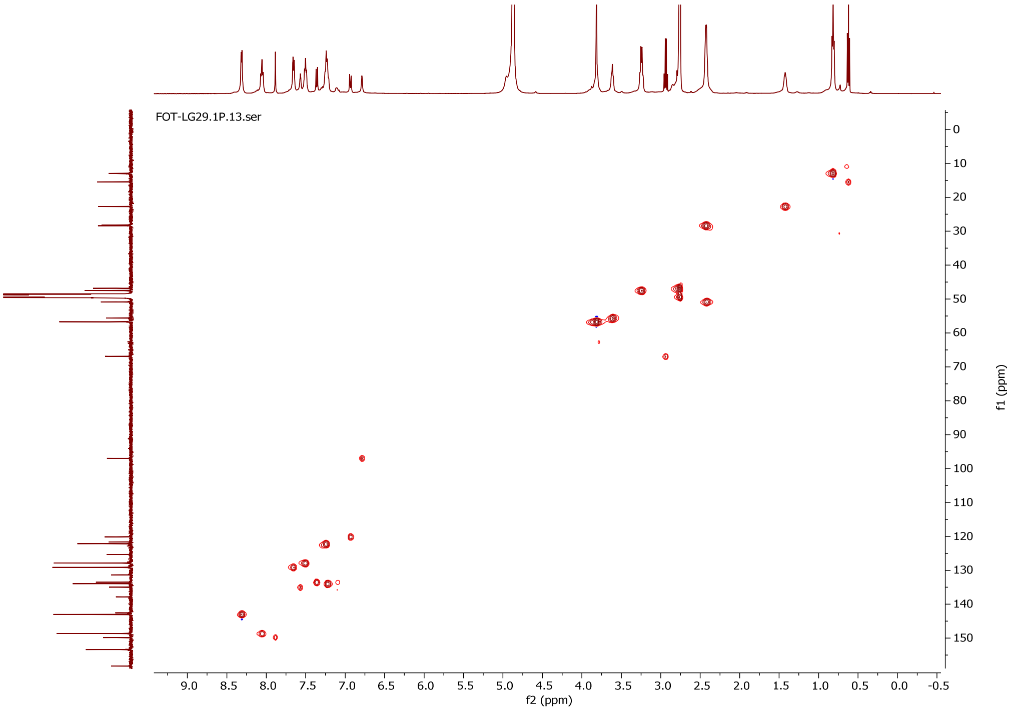

Figure S4. ^{1}H-^{13}C HSQC NMR spectrum of **1**.

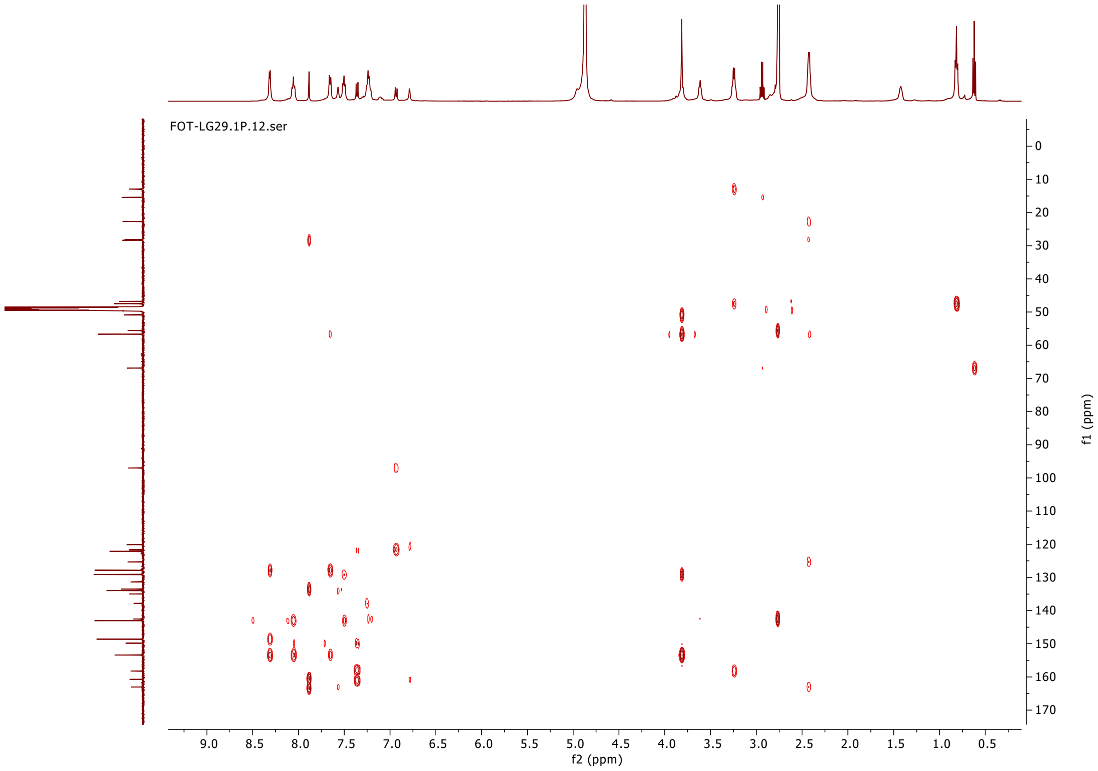

Figure S5. ^{1}H-^{13}C HMBC NMR spectrum of **1**.

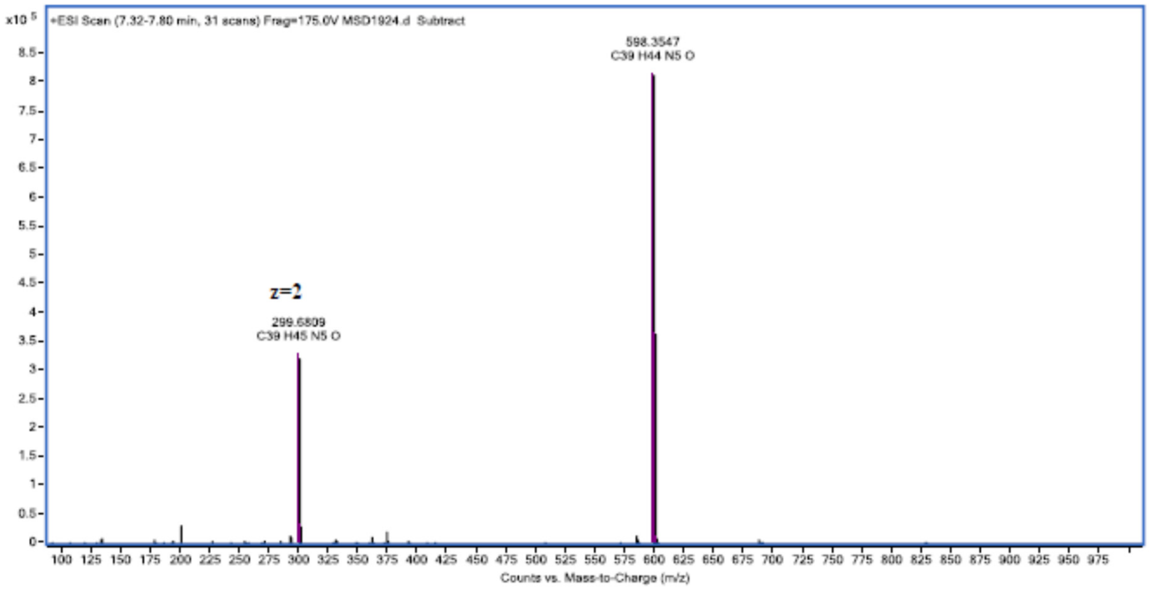

Figure S6. HR-MS spectrum of **1**. Molecular ion was identified at m/z: 598.3547.

Table S1 – Full NMR peak assignment for chemosensor **1**.

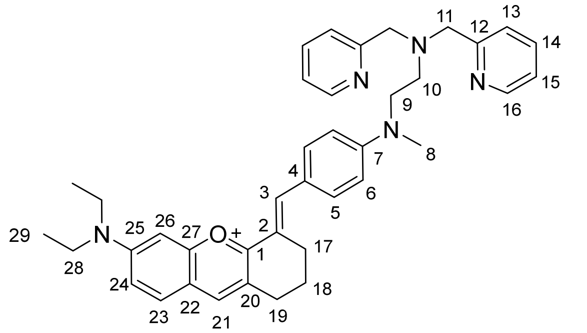

Position	^{1}H	^{13}C
1	-	163.04
2	-	125.33
3	6.79	97.00
4	-	133.51
5	7.23	122.14
6	7.23	133.94
7	-	142.53
8	2.77	46.82
9	3.61	55.58
10	2.43	50.84
11	3.82	56.69
12	-	153.38
13	7.65	129.12
14	8.06	148.64
15	7.50	127.86
16	8.31	143.01
17	2.43	28.17
18	1.43	22.17
19	2.43	38.36
20	-	121.64
21	7.88	149.84
22	-	137.83
23	7.36	131.35
24	6.93	120.12
25	-	158.23
26	7.57	134.97
27	-	160.73
28	3.24	47.48
29	0.82	12.97

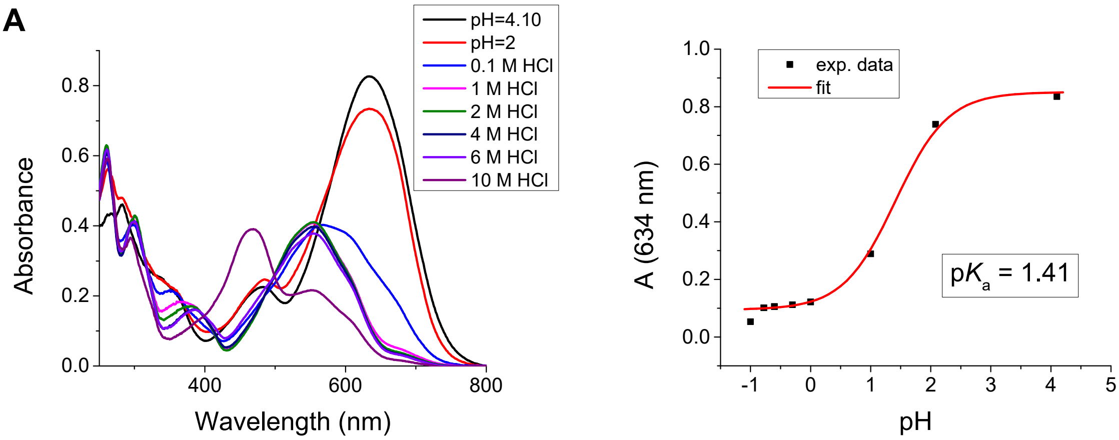

Figure S7. pH titrations of **1** in very acidic conditions: (A) acquired absorption spectra (– 1.7 < pH < 4.1); (B) experimental data points collected at 634 nm, with corresponding fit for the pH equilibrium. Experimental conditions: [**1**] = 3.3 x 10^{-5} M, in H_2O.

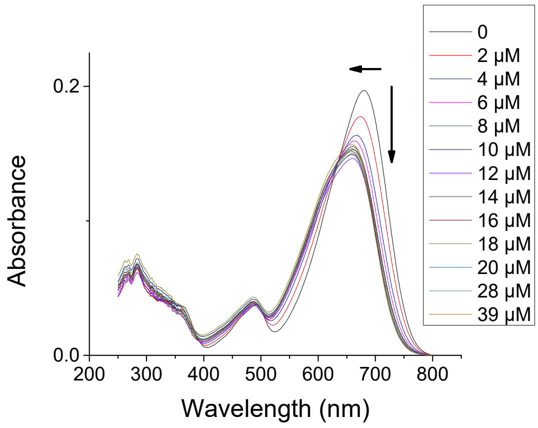

Figure S8. UV-Vis titration of **1** with Zn^{2+}. Conditions: MeOH:MOPS 10 mM pH7 (50:50).

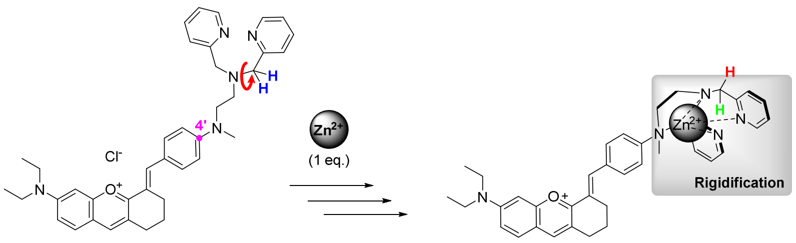

1 [**1**-Zn]$^{2+}$

Figure S9. Representation of the structure rigidification of DPA unit upon complexation with Zn^{2+} metal. Position 4' of the styrylflavylium is signalled in pink. Equivalent protons (blue) become diastereotopic (red and green).

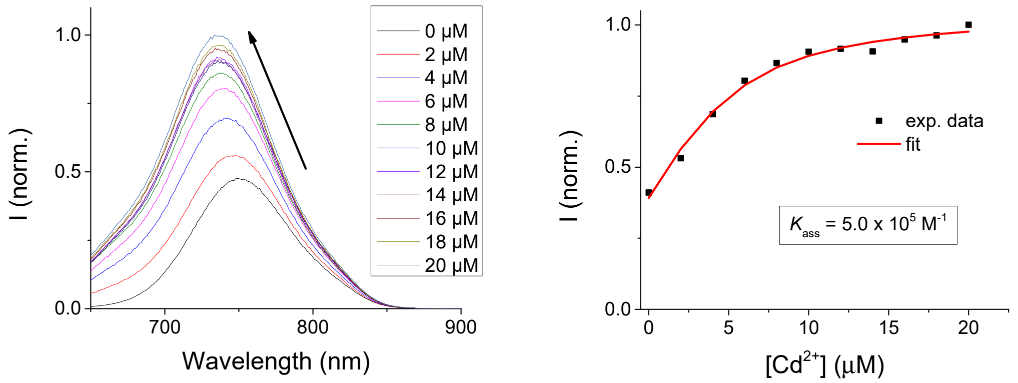

Figure S10. Fluorescence titration of **1** with Cd^{2+}. Conditions: MeOH:MOPS 10 mM pH7 (50:50).

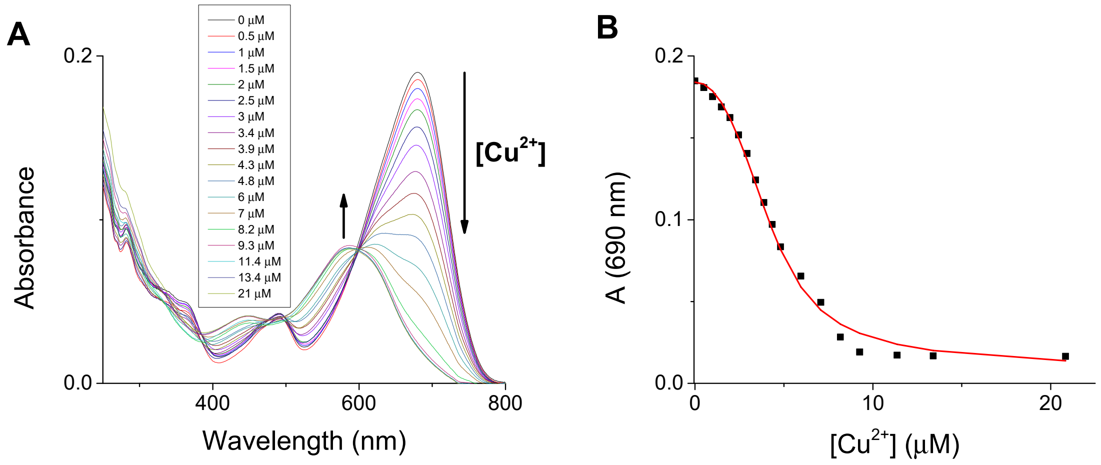

Figure S11. UV-Vis titration of **1** with Cu^{2+}: (A) absorption spectra with increasing Cu^{2+} concentration; (B) recorded absorbance at 690 nm versus Cu^{2+}. Conditions: MeOH:MOPS 10 mM pH7 (50:50).

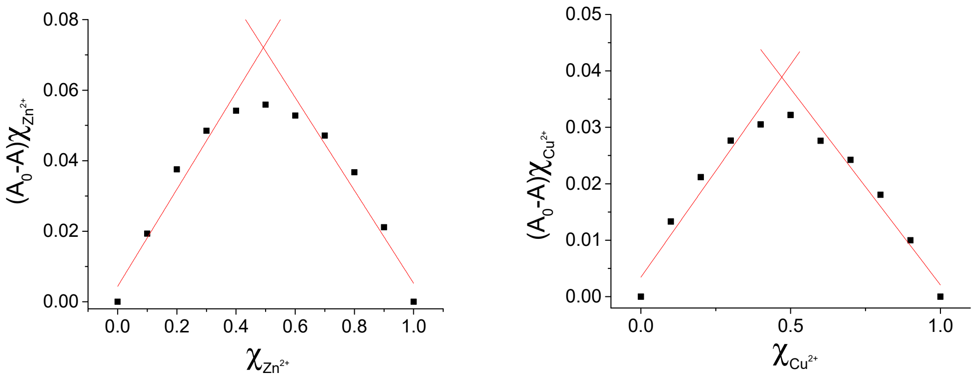

Figure S12. Job's plot of **1** with (A) Zn^{2+} and (B) Cu^{2+}. Intercept between slopes were achieved at molar fraction of metal of 0.49 (Zn^{2+}) and 0.47 (Cu^{2+}). Conditions: MeOH:MOPS 10 mM pH7 (50:50).

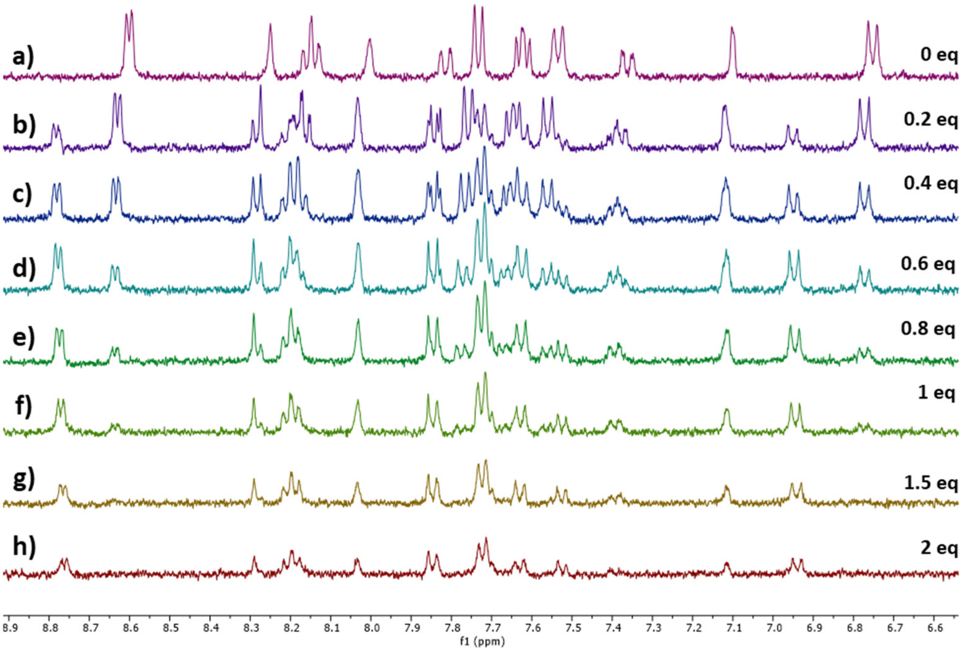

Figure S13. ^{1}H-NMR titration (aromatic protons, 8.9 – 6.6 ppm) of **1** (0.78 mM) with Zn^{2+}, in a mixture of MeOD:D_2O (1:1) at room temperature, between 0 and 2 equiv. of metal.

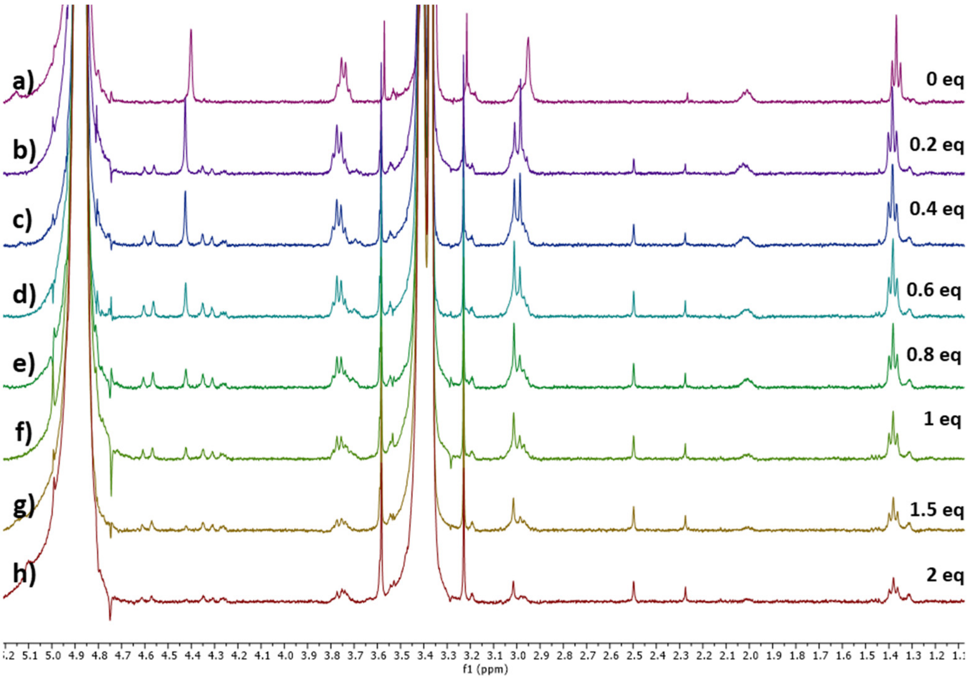

Figure S14. ^{1}H-NMR titration (aliphatic protons, 5.1 – 1.2 ppm) of **1** (0.78 mM) with Zn^{2+}, in a mixture of MeOD:D_2O (1:1) at room temperature, between 0 and 2 equiv. of metal.

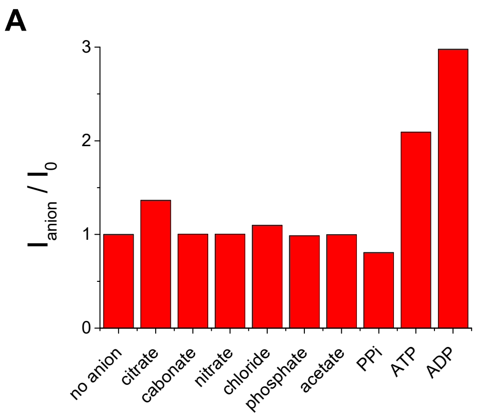

Figure S15. Emission changes of **1**-Zn^{2+} in the presence of different anions. Conditions: MeOH:MOPS 10 mM pH7 (50:50).

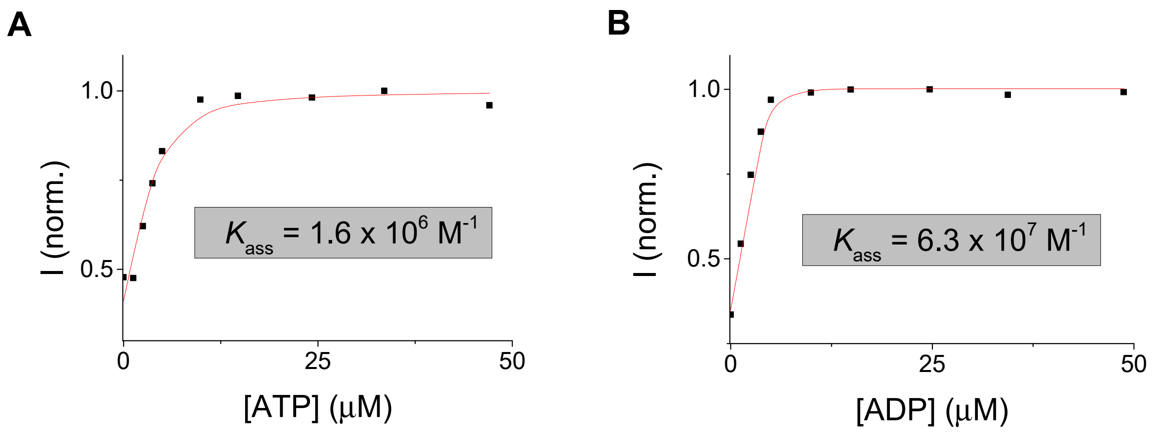

Figure S16. Intensity changes of **1**-Zn^{2+} with ATP (A) and ADP (B) concentration (black dots) with the corresponding fitting curve (red line). Conditions: MeOH:MOPS 10 mM pH7 (50:50); λ_{exc} = 680 nm.

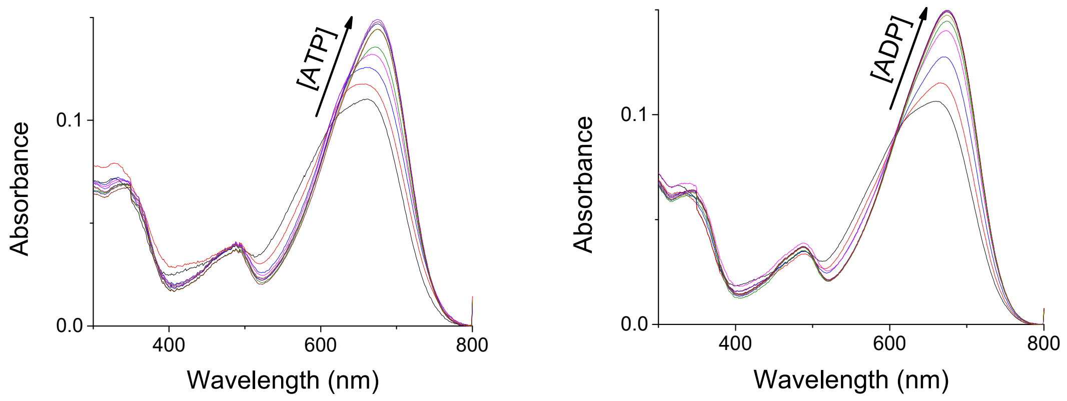

Figure S17. UV-Vis absorption spectra of **1**-Zn^{2+} with increasing concentration of (A) ATP and (B) ADP. Conditions: MeOH:MOPS 10 mM pH7 (50:50).

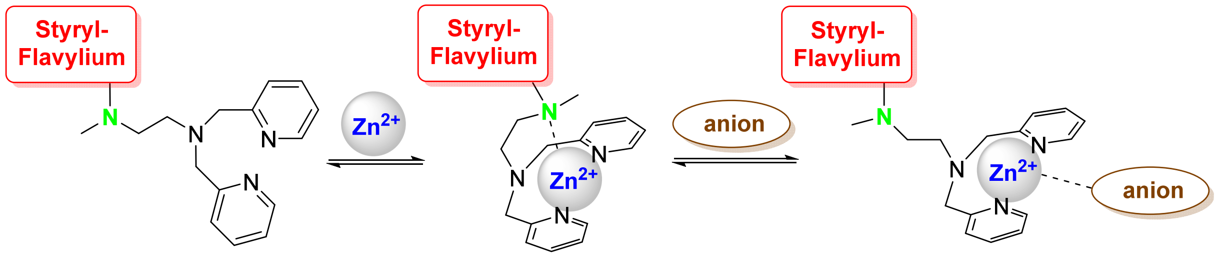

Figure S18. Proposed mechanism for sequential binding of Zn^{2+} and anion, with consequent displacement of the 4'-nitrogen from the styryl flavylium (green) upon anion binding.

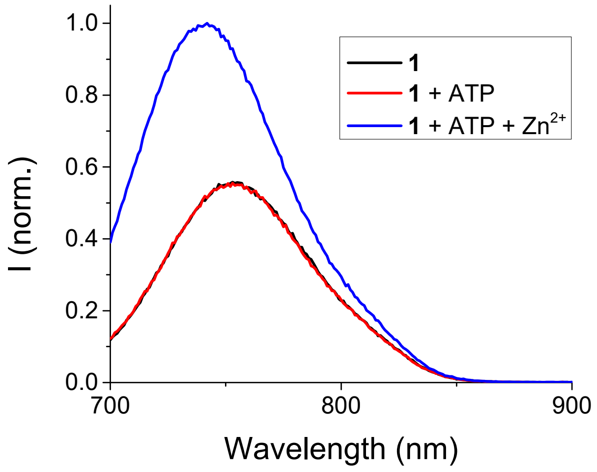

Figure S19. Emission spectra of **1** (black), upon sequential addition of 5 eqs. ATP (red) and 2 eqs. Zn^{2+} (blue).

Mathematical deduction of formulae for fitting the experimental data from titration of chemosensor **1** with Cu(II):

The equilibria between chemosensor **1** and Cu^{2+} is defined by the generic equations (S1) and (S2)

$$K_1 = \frac{[HG]}{[H][G]} \qquad (S1)$$

$$K_2 = \frac{[H_2G]}{[H][HG]} \qquad (S2)$$

where:

H = chemosensor **1** (host)

G = Cu^{2+} ions (guest)

The mass balance from species comprising H and G are described in equations (S3) and (S4):

$$[H]_O = [H] + [HG] + 2[H_2G] \quad (S3)$$

$$[G]_O = [G] + [HG] + [H_2G] \quad (S4)$$

Combination of these four equations leads a third degree equation (S5):

$$A[H]^3 + B[H]^2 + C[H] + D = 0 \qquad (S5)$$

where:

$$A = K_1K_2$$

$$B = 2K_1K_2[G]_O - K_1K_2[H]_O + K_1$$

$$C = K_1[G]_O - K_1[H]_O + 1$$

$$D = -[H]_O$$

with $[H]_0$ and $[G]_0$ representing the total concentrations of chemosensor **1** and Cu^{2+} in solution, respectively.

The best root for this polynomial was determined using Newton's method, which was latter optimized by the *Solver* Add-In from *Microsoft Excel*, by fitting to the experimental data (UV-Vis absorption or emission intensity).
